# Supplementary figures and images for: 1.7 GHz long-term evolution radiofrequency electromagnetic field with stable power monitoring and efficient thermal control has no effect on the proliferation of various human cell types
Source: PLoS One. 2024 May 7;19(5):e0302936. doi: 10.1371/journal.pone.0302936 (PMC11075873; doi:10.1371/journal.pone.0302936)

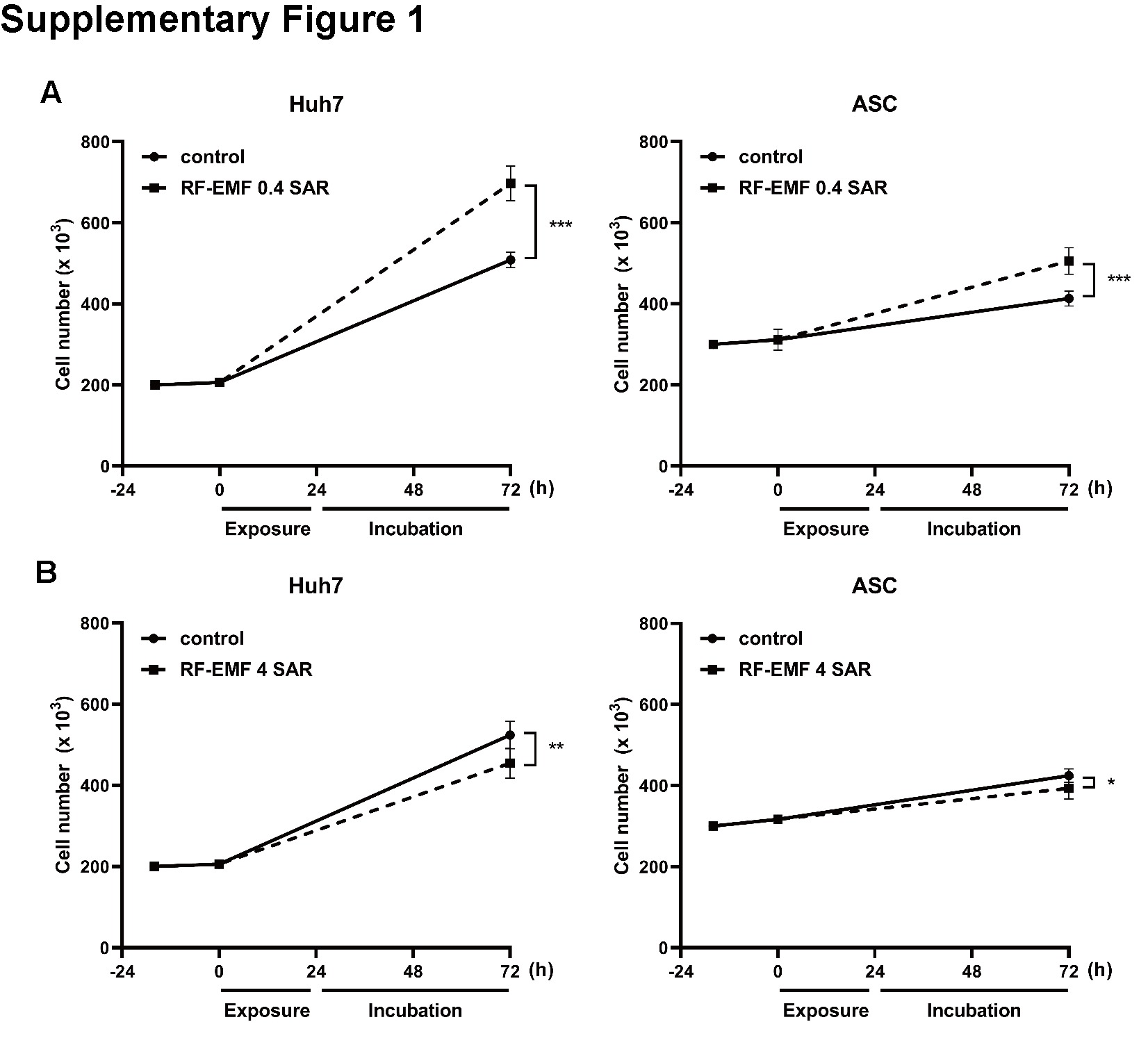

Supplement: S1 Fig — The equal number of ASCs and Huh7 cells was seeded for 16 h, exposed to the SAR of (A) 0.4 W/Kg or (B) 4 W/Kg 1.7 GHz LTE RF-EMF for 24 h, and further incubated for 48 h without RF-EMF exposure. After incubation, the cells were counted using a cell counter and plotted. Three independent experiments were performed, and the cell number is presented as mean ± SD. P < 0.001 (***), P < 0.05 (*). (TIF) [file pone.0302936.s001.tif]

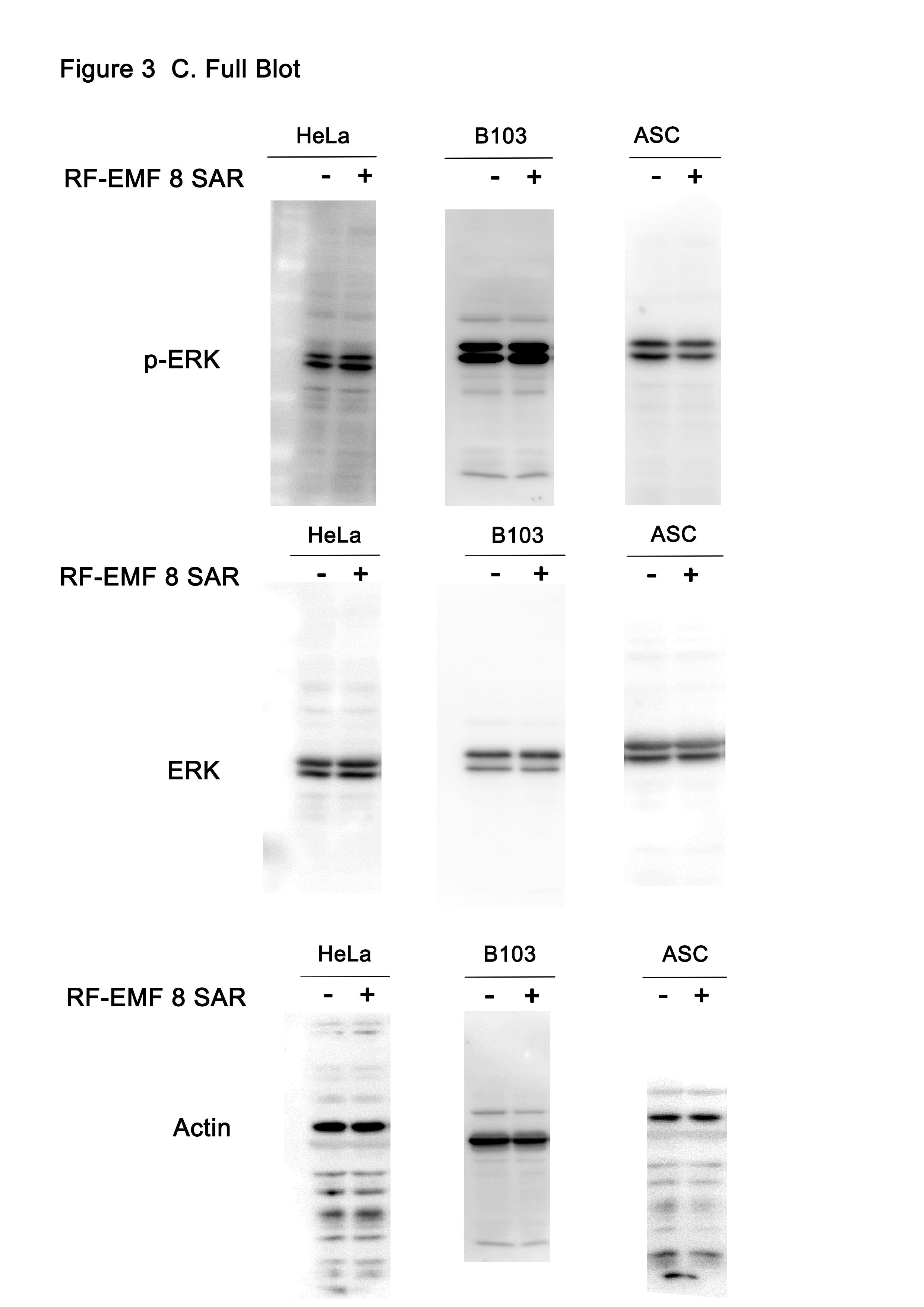

Supplement: S2 Fig — (TIF) [file pone.0302936.s002.tif]

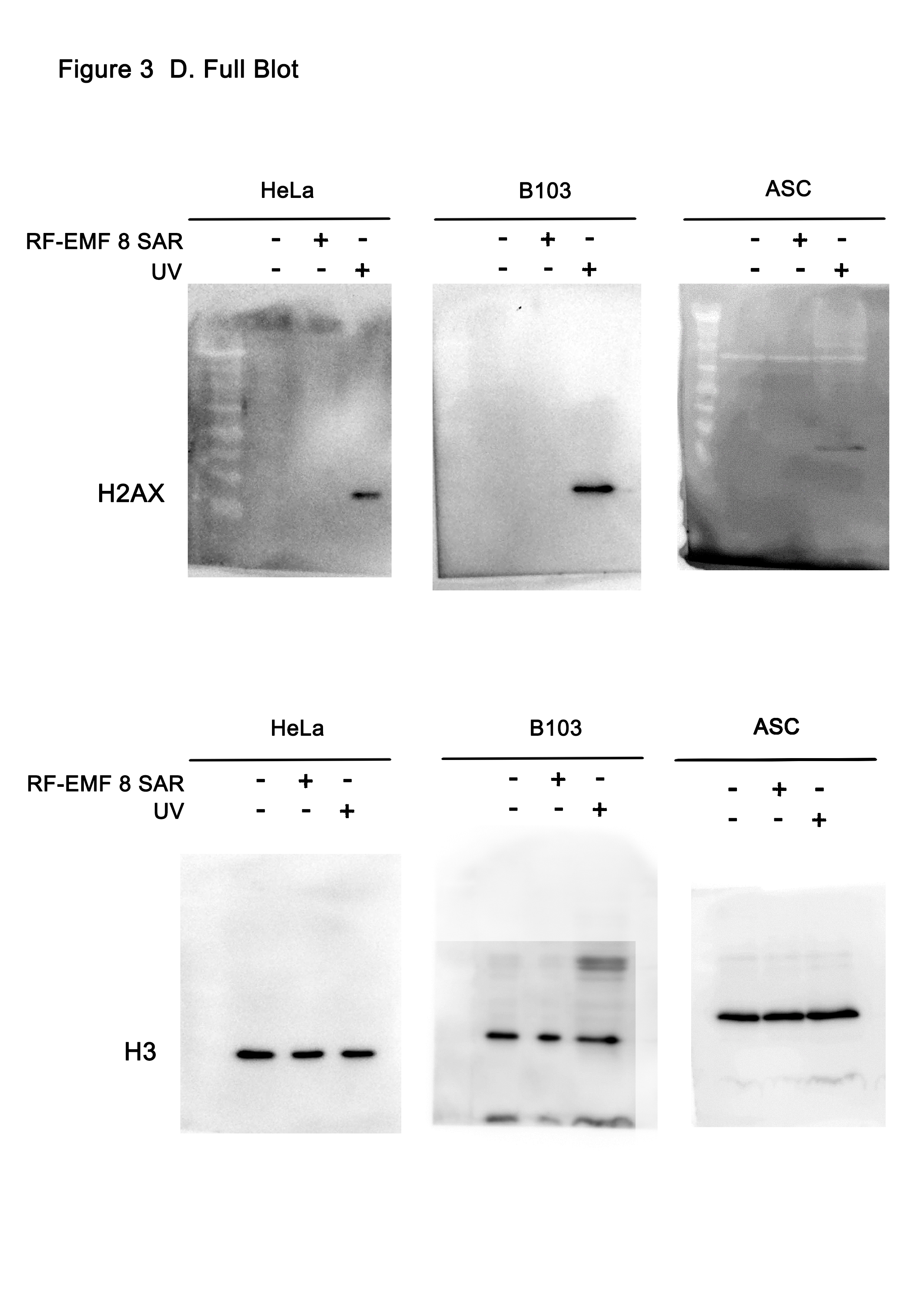

Supplement: S3 Fig — (TIF) [file pone.0302936.s003.tif]
